# Pedunculated Cecal Lipoma Causing Colo-Colonic Intussusception: A Rare Case Report

**DOI:** 10.1155/2012/279213

**Published:** 2012-12-05

**Authors:** Stefanos Atmatzidis, Grigoris Chatzimavroudis, Aristidis Patsas, Basilis Papaziogas, Spiros Kapoulas, Stelios Kalaitzis, Ananias Ananiadis, John Makris, Konstantinos Atmatzidis

**Affiliations:** 2nd Surgical Department, School of Medicine, Aristotle University of Thessaloniki, G. Gennimatas General Hospital, Ethnikis Aminis 41, 54635 Thessaloniki, Greece

## Abstract

Colonic lipomas are uncommon nonepithelial neoplasms that are typically sessile, asymptomatic and incidentally found during endoscopy, surgery, or autopsy. We present a very rare case of a 34-year-old female patient with symptomatic pedunculated cecal lipoma causing intermittent colo-colonic intussusception. Despite adequate imaging studies, definite preoperative diagnosis was not established and the patient underwent exploratory laparotomy. Intraoperatively, intussusception of the cecum into the ascending colon was found and right hemicolectomy was performed. Macroscopic assessment of the resected specimen showed the presence of a giant cecal pedunculated polypoid tumor with features of lipoma, causing intussusception. Histopathological examination confirmed the diagnosis of pedunculated cecal lipoma.

## 1. Introduction

Gastrointestinal lipomas are uncommon nonepithelial neoplasms that can be found anywhere along the entire length of gastrointestinal tract. Typically, they are asymptomatic and incidentally found during endoscopy, surgery, or autopsy. Only 25% of them develop symptoms, especially when their diameter exceeds 2 cm. Colonic lipoma typically presents as a sessile polypoid mass. Infrequently, lipomas of the colon are pedunculated, with ulcerated or necrotic overlying mucosa. We present an extremely rare case of a symptomatic pedunculated lipoma of the cecum with ulcerated mucosa causing intermittent colo-colonic intussusception that was surgically resected.

## 2. Case Report

**Figure 1:**
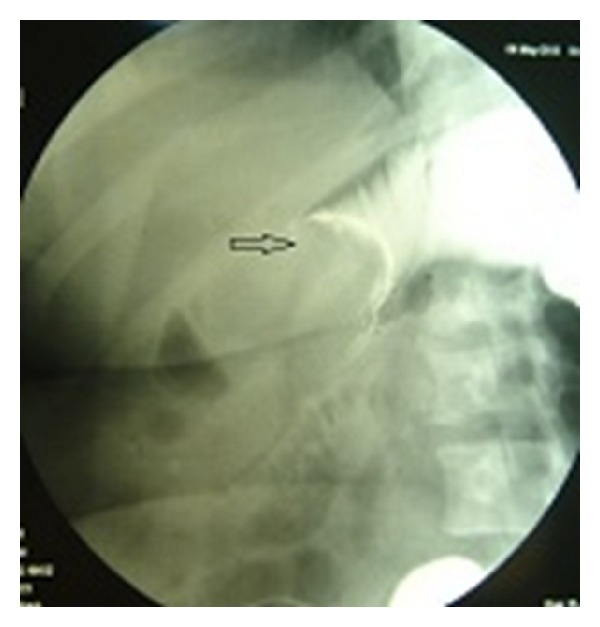
Double-contrast barium enema examination showing an ovoid filling defect (arrow) in the cecum and ascending colon.

**Figure 2:**
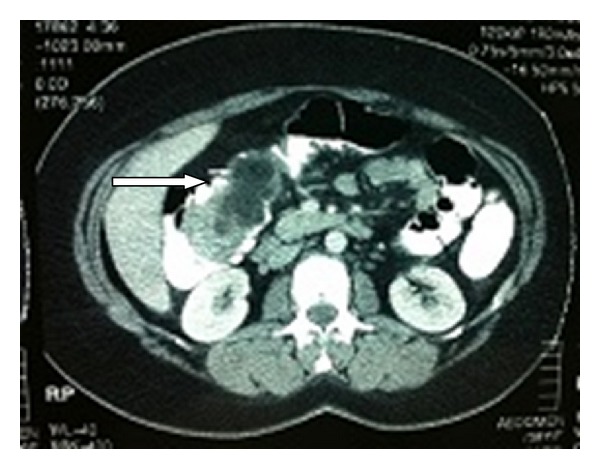
Contrast-enhanced abdominal CT revealing the presence of an ovoid endoluminal mass extending from the cecum to ascending colon with fatty density (arrow) without extramural extension.

**Figure 3:**
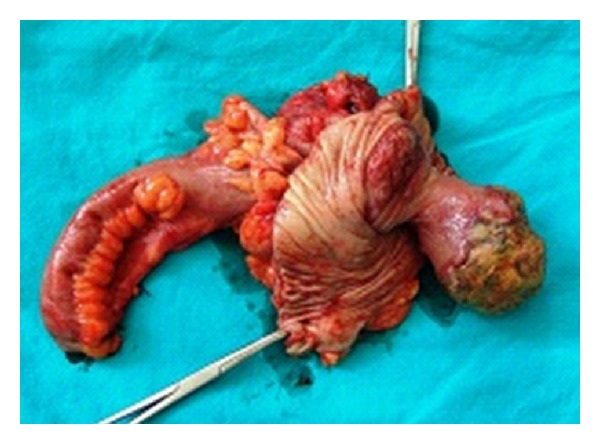
Macroscopic picture of the resected specimen showing the presence of a round pedunculated cecal polypoid tumor with ulcerated overlying mucosa.

A 34-year-old female patient was admitted to our department suffering from intermittent diffuse abdominal pain over the last two months. Her medical history was unremarkable. On examination the abdomen was mildly distended, with rebound percussion tenderness in the right iliac fossa. Routine blood tests were within normal ranges. Double-contrast barium enema examination revealed an ovoid filling defect in the cecum and ascending colon (Figure 1). Subsequently, colonoscopy was performed which, however, was incomplete as the colonoscope could not be passed beyond the hepatic flexure. Contrast-enhanced abdominal computed tomography (CT) showed the presence of an ovoid endoluminal mass extending from the cecum to ascending colon with fatty density, without extramural extension (Figure 2). The patient was transferred to the operating room for exploratory laparotomy, which revealed the presence of a colo-colonic intussusception in the right colon. Due to the high risk of malignant disease, reduction was not attempted and right hemicolectomy was performed. Macroscopic assessment of the resected specimen showed the presence of a round pedunculated cecal polypoid tumor of 6 × 5 × 4,5 cm in size with the features of lipoma, causing intussusception of the cecum into the ascending colon (Figure 3). Histopathology of the resected lesion confirmed the diagnosis of a pedunculated submucosal cecal lipoma with ulcerated overlying mucosa. The postoperative course of the patient was uneventful and she was discharged from the hospital on the 8th postoperative day in good condition.

## 3. Discussion

Colonic lipomas are rare nonepithelial neoplasms with an incidence ranging from 0.035% to 4.4% in relation to all polypoid lesions of large intestine [1]; however, they are the most common tumors of mesenchymal origin of the gastrointestinal tract. Colonic lipoma typically presents as a sessile polypoid mass, arising from the submucosa with an intact mucosa. Infrequently, lipomas of the colon are pedunculated, with ulcerated or necrotic overlying mucosa. They usually involve right colon and are mainly asymptomatic. Occasionally they may present with abdominal pain, rectal bleeding, intermittent diarrhea, obstruction, or rarely with intussusception as in our case [2]. Recently, Paškauskas et al. reviewing the English-language publications of colonic lipomas causing intussusception found less than 50 cases [3]; of these, only in four the intussuscepted segment was cecum-ascending colon. Imaging modalities can contribute to the preoperative diagnosis of colonic lipomas. Barium enema usually reveals a filling defect; however this finding is nonspecific of colonic lipoma or any other type of colonic neoplasm. CT can be diagnostic because typically these tumors have characteristic fatty densitometric values (−40 till −120 Hounsfield units) [4]. However, occasionally colon lipomas might have atypical CT presentation, especially when intussuscepted, due to varying degrees of infarction/fat necrosis. In such cases or when prominent fibrous septa and nodularity are evident, the most imperative differential diagnosis is liposarcoma [5]. Colonoscopy can usually distinguish colonic lipomas from cancer and other neoplasias, especially when the overlying mucosa is intact. However it should be kept in mind that in few cases, accurate preoperative diagnosis can be difficult.

Treatment options of colonic lipomas are various and include endoscopic and surgical procedures. Endoscopic resection is generally recommended for lipomas with a diameter smaller than 2 cm or pedunculated lipomas with thin stalk [6], as in these cases the risk of complications following endoscopic resection is considerably low. However according to Katsinelos et al., if a lipoma is sessile or broadly-based, even if its diameter is less than 2 cm, endoscopic removal is risky because the adipose tissue is an inefficient conductor for electric current and may result in a significantly high rate of complications like perforation or hemorrhage [7]. The majority of authors recommend surgery as the standard method of treatment for every colonic lipoma greater than 2 cm in size [3, 6]. Surgical treatment includes resection, colotomy with local excision, limited colon resection, segmental resection, hemicolectomy, or subtotal colectomy; the choice of any of the above-mentioned surgical interventions mainly depends on the lipoma size, location, and the presence or absence of definite preoperative diagnosis or disease complications [6]. During last years a few selected cases of successful laparoscopic resection under colonoscopic guidance of symptomatic colonic lipomas have been reported [8, 9]. A topic for debate is whether resection should be performed with or without prior reduction of the intussuscepted lesion. Though various views have been expressed, the most widely accepted one is that in most cases of ileocolic and colocolic intussusception, the procedure of choice should be primary resection without reduction due to the high risk of underlying malignancy [10].
